# The molecular mechanism of Gaucher disease caused by compound heterozygous mutations in *GBA1* gene

**DOI:** 10.3389/fped.2023.1092645

**Published:** 2023-01-26

**Authors:** Qi Liu, Zongrui Shen, Hong Pan, Shunfei Ma, Fu Xiong, Fei He

**Affiliations:** ^1^ Department of Transfusion, Shaoxing People’s Hospital, Shaoxing, China; ^2^ Department of Medical Genetics, School of Basic Medical Sciences, Southern Medical University, Guangzhou, China; ^3^ Experimental Education/Administration Center, School of Basic Medical Science, Southern Medical University, Guangzhou, China; ^4^ Department of Hematology, Shaoxing People’s Hospital, Shaoxing, China

**Keywords:** Gaucher disease, *GBA1*, c.1240G > C, c.1342G > C, cell experiment

## Abstract

Gaucher disease (GD, ORPHA355) is a rare autosomal recessive genetic disease caused by mutations in *GBA1*, which encodes the lysosomal enzyme glucocerebrosidase (GCase). Here, we report a patient with GD who carried the heterozygous c.1240G > C (p.Val414Leu) mutation and the heterozygous pathogenic c.1342G > C (p.Asp448His) mutation in *GBA1*. Bioinformatics analysis suggested that the two mutations are pathogenic. Functional studies showed that *GBA1* mRNA and GCase protein levels of mutant types were significantly less than the wild-type. In the cell lysates, the two mutations of *GBA1* c.1240G > C and c.1342G > C caused a decreased GCase concentration, while the two mutations did not change the distribution in the cell. The pathogenicity of the compound heterozygous mutations was verified. Early diagnosis and treatment can improve the quality of life and prevent unnecessary procedures in patients with GD.

## Introduction

Gaucher disease (GD, ORPHA355) is a rare autosomal recessive genetic disease, affecting 1/50,000 to 1/100,000 individuals in the general population. However, its frequency is as high as 1:800 in Ashkenazi Jews (1). GD is caused by mutations in *GBA1* (OMIM #606463), which encodes the lysosomal enzyme glucocerebrosidase (GCase) (2). To date, more than 500 mutations have been described in the *GBA1* gene, including single base changes, splicing alterations, partial and total deletions, insertions, and rearrangements (3, 4). GD is categorized into three clinical phenotypes based on the presence or absence of neurological involvement (5). A diagnosis of GD is established by the detection of GCase or by molecular genetics testing (6).

In this study, we report a patient with GD who carried the c.1240G > C (p.Val414Leu) mutation and c.1342G > C (p.Asp448His) mutation in *GBA1*. The pathogenicity of the compound heterozygous mutation was verified with cell experiments.

## Materials and methods

### Patient

The diagnosis was based on clinical data as well as reduced GCase activity and molecular analysis. This study conformed to human research guidelines stated in the Declaration of Helsinki and written informed consent was obtained from all subjects. The female patient was a 62-year-old who first visited Shaoxing People's Hospital because of massive splenomegaly and thrombocytopenia with no neurological impairment.

### Bioinformatics analysis

The three-dimensional (3D) protein structures of the wild-type and mutant GCase were predicted using I-TASSER (http://zhanglab.ccmb.med.umich.edu/I-TASSER) and *Pymol*. *ClustalX* was used to analyze evolutionary conservation among different paralogs and orthologs.

### DNA extraction, purification, and single-gene sequencing

DNA was extracted from fresh blood using a standard phenol-chloroform extraction method after digestion with proteinase K to ensure that the DNA quality and quantity met the experimental requirements. Specific primers were used to amplify the 8–11 exon of *GBA1* gene. The forward and reverse sequences were as follows: forward primer (5′-TGTGTGCAAGGTCCAGGATCAG-3′), reverse primer (5′-ACCACCTAGAGGGGAAAGTG-3′). Sanger sequencing was performed by Sangon Biotech (Shanghai, China). The sequences obtained were aligned to the available reference sequence in The National Centre for Biotechnology Information GeneBank database to detect variants.

### Plasmid construct

The length of the *GBA1* cDNA is approximately 1.6 kb. Total RNA from peripheral blood lymphocytes of healthy family members was extracted with Trizol reagent (Invitrogen, Carlsbad, CA, United States) and was reverse transcribed into first-strand cDNA with the HiScript II 1st Strand cDNA Synthesis Kit (Vazyme, Jiangsu, China) according to the manufacturer's instructions. The wild-type *GBA1* cDNA was cloned using primers containing the XhoI and EcoRI restriction sites. The forward and reverse sequences were as follows: forward primer (5′-CCGCTCGAGAGATGGAGTTTTCAAG-3′),reverse primer (5′-GGAATTCCACTGGCGACGCCACAG-3′). To clone the verified PCR products into the pEGFP-N1 vector by double digestion with XhoI and EcoRI. *GBA1* mutations were introduced into the wild-type plasmid by PCR-based site-directed mutagenesis to obtain the pEGFP-N1-*GBA1*-MUT vector (c.1240G > C:forward primer: 5′-AACCTCCTGTACCATCTGGTCGGCTGGACC-3′; reverse primer: 5′-GGTCCAGCCGACCAGATGGTACAGGAGGTT-3′; c.1342G > C: forward primer: 5′-GTAGACATCACC AAGCACACGTTTTACAAA-3′; reverse primer: 5′-TTTGTAAAACGTGTGCTTGGTGATGTCTAC-3′) with the Phanta Max Super-Fidelity DNA polymerase and confirmed by sequencing. The plasmids were extracted with the endotoxin-free plasmid mini-extraction kit (Tiangen, Beijing, China).

### HEK 293 T cell line culture and transient transfection

HEK 293 T cells were grown in Dulbecco's Modified Eagle Medium (Gibco, NY, United States) supplemented with 10% fetal bovine serum (Gibco), 100 U/ml penicillin, and 100 U/ml streptomycin at 37 °C in a humidified atmosphere enriched with 5% CO_2_. Cell transfection was performed according to the manufacturer's instructions using Lipofectamine 3,000 transfection reagent (Invitrogen) for the pEGFP-N1 vector, and the *GBA1* wild-type (WT) and mutant-type (MT), respectively. After 48 h transfection, cells were collected for GCase enzyme assays and Western blotting. All transfections were performed in duplicate in three individual experiments.

### Immunofluorescence detection and subcellular localization

The cells were grown on Petri dishes and fixed with cold 4% formaldehyde for 30 min at −20 °C and blocked in 0.2% Triton X-100 for 5 min at room temperature. The fixed cells were separately stained with DAPI to visualize the nuclear DNA. Fluorescence microscopy was carried out with an LSM880 confocal microscope equipped with appropriate filter sets.

### ELISA

When HEK 293 T cells reached 70% confluence in 6-well plates, 2.5 µg pEGFP-N1-*GBA1*-WT and MT were transfected into the cells using Lipofectamine 3,000 transfection reagent (Invitrogen). The cells were collected by centrifugation and washed with pre-chilled PBS after 48 h transfection, then 500 µl PBS was added to resuspend the cells, and the total cellular protein was extracted by repeated freezing and thawing. GCase concentrations were detected using the Human Glucosylceramidase (GCase) ELISA Kit (CUSABIO).

### Gcase enzyme activity assay

When HEK 293 T cells reached 50% confluence in 6-well plates, 2.5 µg pEGFP-N1-*GBA1*-WT and MT were transfected into the cells using Lipofectamine 3,000 transfection reagent (Invitrogen). 48 h after transfection, cells were collected and washed with cold PBS (Sigma–Aldrich), and the total cell protein was extracted by repeated freezing and thawing before resuspension of the cells in 150 µl PBS containing 1% PMSF. Cell debris was removed by centrifugation, and cell lysates were quantified using the Pierce™ Rapid Gold BCA Protein Assay Kit (Thermo Fisher Scientific). 15 μg protein was mixed with 20 ul 4-methylumbelliferyl-β-glucosidase substrate, incubated at 37 °C for 2 h, and 200 ul glycine buffer was added to terminate the reaction. Sample 4-methylumbelliferone fluorescence intensity was measured using a microplate reader at an excitation wavelength of 355 nm and an emission wavelength of 460 nm.

### RNA expression

Total RNA was extracted with TRIzol reagent (Invitrogen) from the transfected cells. RT-PCR was performed using the HiScript II 1st Strand cDNA Synthesis Kit (Vazyme, Nanjing, China). *GBA1* mRNA expression levels were analyzed using the ChamQ SYBR qPCR Master Mix (Vazyme) with the specific primers as follows: forward primer5′-TCCCAAGCCTTTGAGTAGGG-3′, reverse primer 5′-GCCGAAGCTTTTAGGGATGC-3′. Gene expression levels were calculated using the 2^−ΔΔ^CT method. Expression levels of the target gene was normalized to that of glyceraldehyde 3-phosphate dehydrogenase (*GAPDH*) with the specific primers: forward primer 5′-GTGAAGGTCGGAGTCAACG-3′, reverse primer 5′-TGAGGTCAATGAAGGGGTC-3′.

### Western blotting

The transfected cells were lysed using cell lysis buffer for Western blot (Beyotime, Beijing, China) containing 1% PMSF. Protein loading buffer was added to the cell lysates before boiling for 5 min. Protein (25 µg) was loaded for electrophoresis on 10% SDS-PAGE gels and transferred to PVDF membranes. Then, the membranes were blocked with 5% non-fat milk in TBS containing 0.1% Tween 20 for 2 h at room temperature and incubated with antibodies against EGFP (1:2,000, Proteintech, Wuhan, China) at 4 °C overnight. Finally, the membranes were incubated with a secondary goat anti-mouse IgG (1:5,000) for 2 h at room temperature. The signals were detected using the ECL Western blotting detection reagent.

### Statistical analysis

The results are presented as means ± standard deviation of at least three independent biological replicates. At least three technical replicates were used per experiment. The differences between the WT and MT enzyme activities were analyzed using a *t*-test with GraphPad Prism 5 (GraphPad Software Inc., CA, United States). A *p*-value of ≤0.05 was considered statistically significant.

## Results

### Clinical phenotype

**Figure 1:**
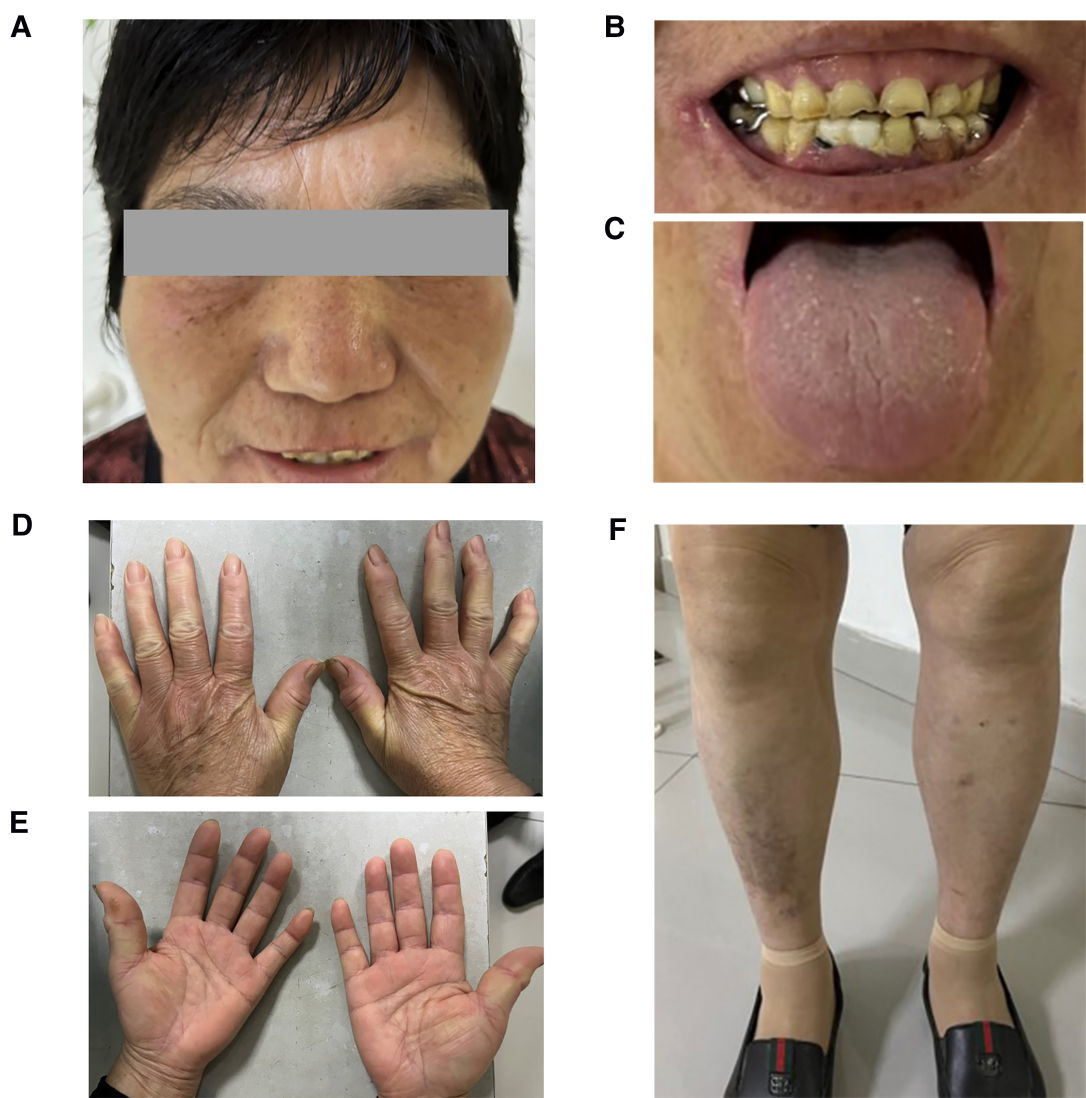
The patient's clinical manifestations.

The patient was diagnosed with Gaucher disease type I. She was conscious and had no anemic appearance and no ecchymosis or petechiae on the skin of limbs (Figure 1). There was no significant superficial lymphadenopathy. Her breath sounds were clear, without dry or wet sounds. The heart rate was 65 beats/min and was rhythmical. The abdomen was flat and soft, without pressure pain or rebound pain. There was no percussion pain in either kidney and no swelling in the lower limbs. The neurological examination was normal. Routine blood tests showed normal hemoglobin and mild thrombocytopenia. The GCase activity was lower than the reference value. The pathological results showed that the proportion of hematopoietic tissue and fat in the bone and bone marrow was essentially normal. The proportion of granulocytes and erythrocytes in the hematopoietic tissue was approximately normal, and megakaryocytes were occasionally seen. The bone density was generally decreased in the lower and middle femur, knee, pelvis, and both hips. Hyperosteogeny was seen in the knee and both hips. There were no other skeletal abnormalities, fractures, pain and bruising. The physical examination results of the patient are shown in Table 1.

**Table 1:** Physical examination results of the patient.

Examination item	Test value	Reference value
Leucocytes	4.08 × 10^9^/L	(3.50–9.50) × 10^9^/L
Lymphocyte percentage	19.4% ↓	(20.0–50.0)%
Lymphocyte counts	0.79 × 10^9^/L ↓	(1.1–3.2) × 10^9^/L
Platelets	58.0 × 10^9^/L ↓	(125–350) × 10^9^/L
Immunoglobulin G	24.13 g/L ↑	(7.00–17.00) g/L
Immunoglobulin A	4.45 g/L ↑	(0.70–4.29) g/L
Immunoglobulin M	3.50 g/L ↑	(0.29–3.44) g/L
γ-pancreatic acyltransferase	47.2 U/L ↑	(7.0–45.0) U/L
Blood urea nitrogen	3.61 µmol/L	(2.86–8.20) µmol/L
Creatinine	42.4 µmol/L ↓	(45.0–84.0) µmol/L
GCase activity	2.3 nmol/mg.h ↓	6.5–20.6 nmol/mg.h
Antinuclear antibodies	1:640 ↑	<1:40
Anti–Scl-70 antibodies	8,670 KD	
Liver size	20.7 × 14.8 × 9.9 cm	(25–26) × (15–16) × (5–6) cm
Spleen size	28.5 × 13.2 × 6.7 cm ↑	(10–12) × (6–8) × (3–4) cm

### Identification of *GBA1* mutations

**Figure 2:**
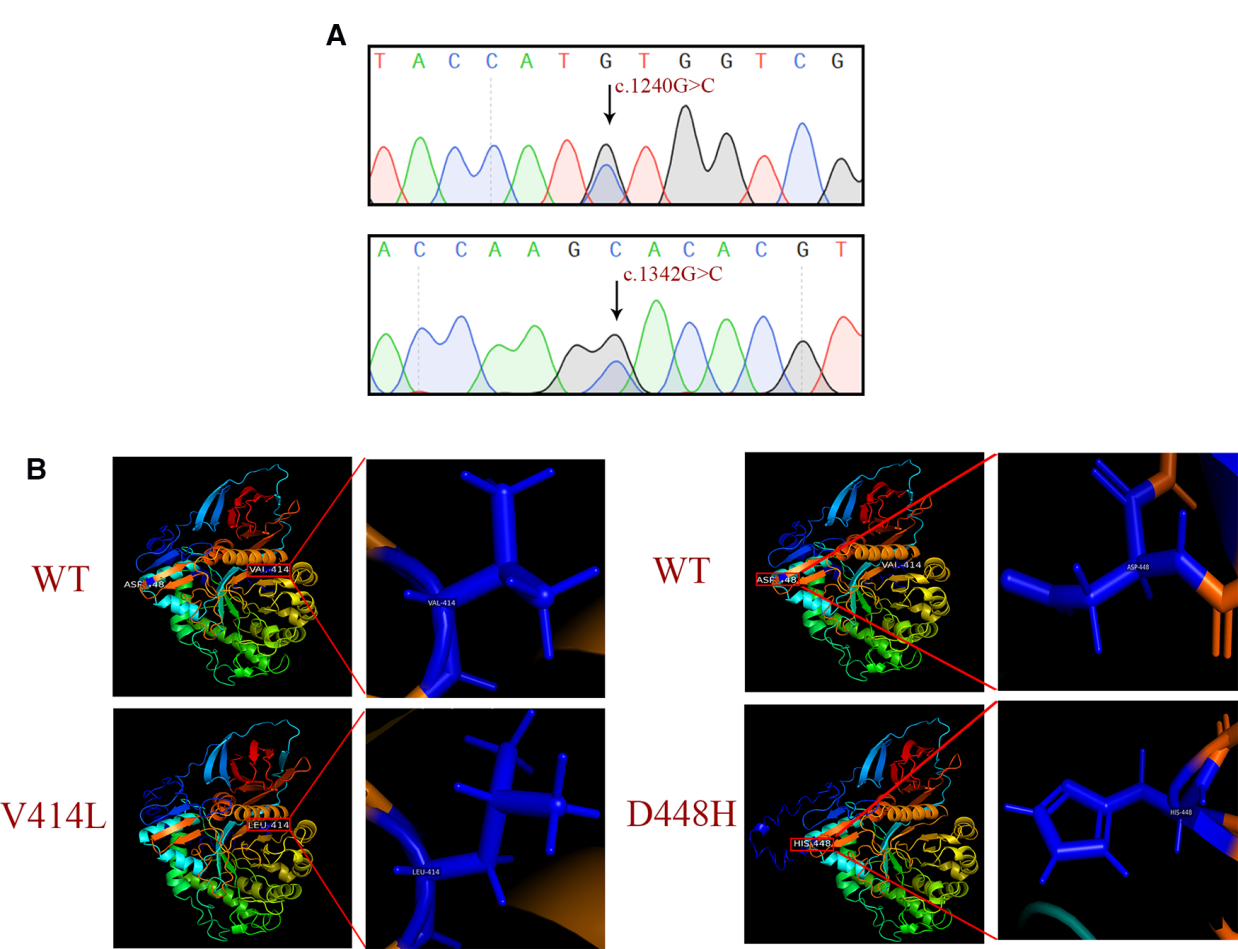
Sequencing chromatogram and bioinformatics analysis. (**A**) Sanger sequencing results of the c.1240G > C (p.Val414Leu) and c.1342G > C (p.Asp448His) mutations. (**B**) Protein structure predicted by I-TASSER.

Guangzhou Women's and Children's Hospital (Guangzhou, China) had previously performed sanger sequencing for 11 exons and the exon-intron junction region of the *GBA1* gene. DNA sequencing revealed a mutation c.1240G > C (p.Val414Leu) and a known pathogenic mutation c.1342G > C (p. Asp448His) (Figure 2A). The mutations were predicted to alter both the secondary structure and the three-dimensional structures of GCase. In addition, the three-dimensional structures of the WT and MT proteins were visually distinct in both the overall and partial views. The c.1240G > C mutation might affect the catalytic function by disturbing the glycosylation process, and the substitution of the bulky phenylalanine with a small leucine residue might bring in a larger side chain that could form steric effects in the catalytic region (Figure 2).

**Figure 3:**
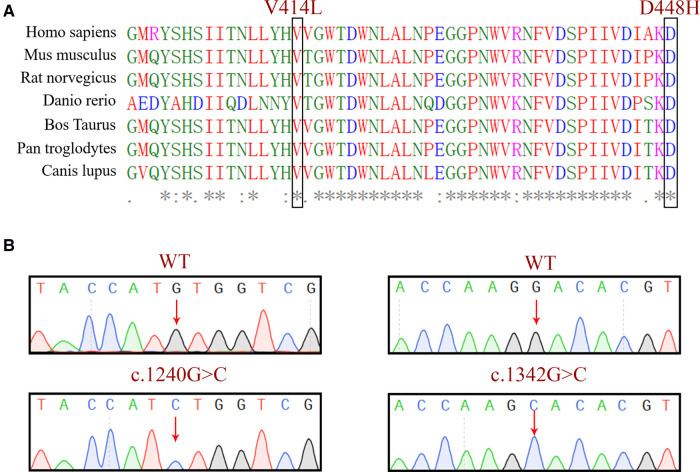
(**A**) Evolutionary conservation analysis of a multiple sequence alignment of the GCase. The comparison between different eukaryotic species, showing that the base pair involving the nucleotide at positions 1,240 and 1,342 in GCase are highly conserved. (**B**) Sequencing validation of the constructed plasmids.

Evolutionary conservation analysis of multiple sequence alignments of the GCase protein and its homologs showed that the amino acid at residues 414 and 448 are evolutionarily conserved (Figure 3A), suggesting that these amino acids may play an important role in the function of GCase. We verified the constructed plasmids with sequencing, and the results are shown in Figure 3B.

### Functional analysis after plasmid transfection

**Figure 4:**
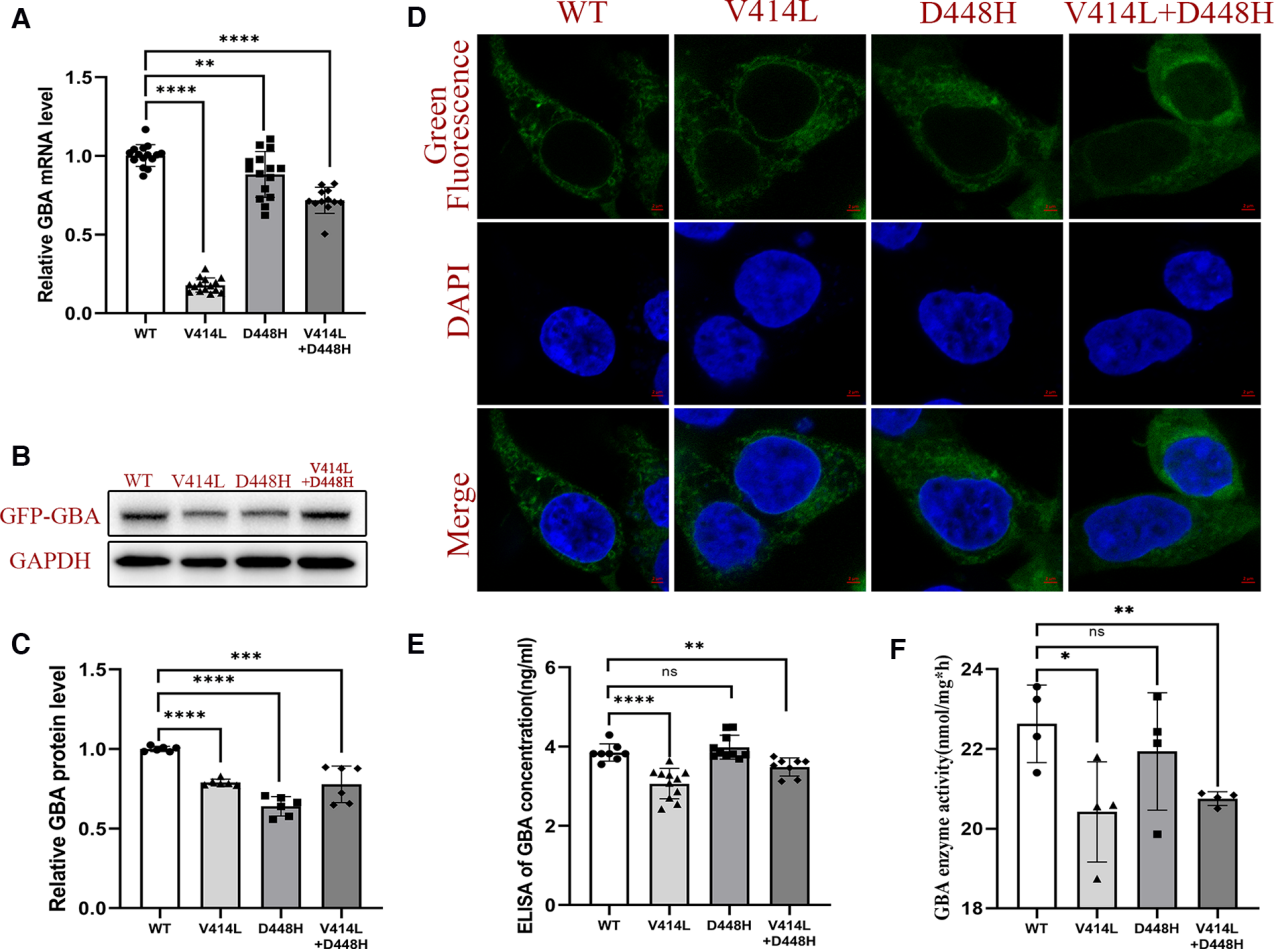
Functional analysis after plasmid transfection. (**A**) mRNA expression level of *GBA1* in HEK293T cells. There was a significant difference in the mRNA level of the wild-type and the mutant. (**B**) Western blotting analysis of GCase expression. (**C**) Protein expression level of GCase in HEK293T cells. Quantitative detection found that the WT *GBA1* was more highly expressed than the MT at both the mRNA and protein levels (*p *< 0.05). (**D**) The subcellular localization analysis showed that both the MT and WT were localized at the cytoplasm. (**E**) The GCase concentration was significantly different between the WT and the V414L mutation or V414L + D448H. (**F**) The GCase enzyme activity assay was significantly different between the WT and the V414L mutation or V414L + D448H.

To further study the functional consequences of both *GBA1* mutation *in vitro*, we examined the expression of *GBA1* using qPCR and Western blotting. Quantitative detection revealed that the WT *GBA1* was more highly expressed than the MT at both the mRNA and protein levels (*p* < 0.05) (Figures 4A–C).

The subcellular localization analysis showed that both the MT and WT were localized at the cytoplasm, suggesting there is no difference in subcellular localization (Figure 4D). We also detected the GCase concentration, and the results showed that there was a significant difference between the WT and the MT with the V414L mutation, or that with V414L + D448H. However, no significant difference was found between the WT and D448H (Figure 4E). The results of GCase enzyme activity assay showed that there also was a significant difference between the WT and the MT with the V414L mutation, or that with the V414L + D448H mutation (Figure 4F).

## Discussion

GD is a rare hereditary lysosomal storage disorder caused by defects in the *GBA1* gene (7). Splenomegaly, bone disease, and thrombocytopenia are the most common characteristics of GD (8). The *GBA1* gene is located in a gene-rich region on chromosome 1q21 and has a highly homologous pseudogene *GBAP1* sequence located 16 kb downstream that shares 96% exonic homology with the functional gene. It is composed of 11 exons. A useful distinction for molecular diagnostic applications is that the pseudogene *GBAP1* has a 55 bp deletion in exon 9. By Sanger sequencing of the exon 8–11 region of DNA from the patient's peripheral blood, we not only confirmed those two heterozygous mutations but also confirmed that both mutations are located in *GBA1* rather than *GBAP1*. Many mutations associated with GD have been reported (4, 9). There are different types of *GBA1* gene mutations and different pathogenic modes, so it is necessary to use appropriate methods to evaluate the pathogenicity of *GBA1* gene mutations (3). Large fragment deletion-insertion mutations can directly affect the process of transcription and translation and are often considered pathogenic. In nonsense mutations, termination codons appear early, potentially leading to premature mRNA degradation or producing truncated proteins that affect its stability and normal function, so these are often considered pathogenic. Missense mutations are more difficult to determine and must be evaluated using biological information and functional studies.

In this study, we identified a heterozygous c.1240G > C (p.Val414Leu) mutation, on which functional studies, including subcellular localization, mRNA level, and protein expression, were performed to confirm its pathogenicity. This mutation has not been previously reported, and the possibility of polymorphism was excluded. This c.1240G > C mutation is predicted to be possibly damaging, and the amino acids are highly conserved in different species, which supports that this mutation is likely to be pathogenic. Another heterozygous pathogenic c.1342G > C (p.Asp448His) mutation, also known as D409H, contributes to α-synuclein accumulation and leads to GCase deficiency. This has been widely reported in at least 33 individuals (10). The allele frequency of this mutation in a cohort of 436 GD patients in Portugal and Spain was 3.3% (11). This variant typically causes a severe form of the disease, and could cause different severity of GD (3).

In vitro functional studies of *GBA1* gene mutations can help us understand the molecular pathogenesis of these mutations and evaluate the pathogenicity of mutations. We found that the decrease in mRNA caused by compound heterozygous mutation was between two simple independent mutations. However, the decrease in protein level was not as obvious as that of the two simple independent mutations. This inconsistency may be due to protein stability and protein-protein interactions. There was no difference in subcellular localization, and the protein seemed to be located more in the nucleus after co-transformation, which may be due to protein degradation slowing down after co-transformation, resulting in slightly more protein. In this study, all results were consistent. At the molecular function level, the V414L mutation was more significantly altered than the V414L + D448H mutaition. Clinically, we observed that people carrying only the V414L heterozygous mutation did not have a severe phenotype. This may be because human have two chromosomes, one from the mother and one from the father. When one of them is mutated, the other can still normally translate proteins to meet body needs. However, when both chromosomes are mutated and the body is unable to translate completely the correct protein, it can lead to severe disease. From the molecular function results presented in this study, we can predict that patients will present with a more distinct disease phenotype when they carry the V414L homozygous mutation.

Genetic analysis can be used to confirm the diagnosis of GD, to help analyze the relationship between clinical phenotype and genotype, to evaluate the prognosis of the disease, and to aid in genetic counseling and prenatal diagnosis. Early diagnosis of GD can be achieved by combining clinical manifestations, laboratory testing, and genetic analyses (12, 13).

Our research provides a reference for the same mutant loci discovered in the future and to avoid duplication of work. On the other hand, With the continuous application of new technology and the deepening of the research, new mutant loci are continuously discovered, which may form a network of *GBA1* gene mutations associated with Gaucher disease and provide new perspectives for further research on the pathogenesis of Gaucher disease.

Multiple studies have implicated heterozygous *GBA1* mutations as a major genetic risk factor for Parkinson's disease (14, 15). Therefore, it is necessary to identify the diagnosis with a combination of clinical and biochemical examinations.

## Conclusion

The mutation spectrum of *GBA1* exhibits ethnic and regional disparity in Asian patients. Our study provides evidence that V414L and D448H in *GBA1* gene can be pathogenic and lead to GD. The findings expand the phenotypic and mutation spectra, and will benefit further genetic studies of the *GBA1* gene in patients with GD.

## Data Availability

The original contributions presented in the study are included in the article/Supplementary Material, further inquiries can be directed to the corresponding author/s.
